# The evolution of preference strength under sensory bias: a role for indirect selection?

**DOI:** 10.1002/ece3.273

**Published:** 2012-07

**Authors:** Alicia M Frame, Maria R Servedio

**Affiliations:** Department of Biology, University of North Carolina at Chapel HillCB 3280, Chapel Hill, North Carolina 27599

**Keywords:** Evolution, female preference, indirect selection, male color polymorphisms, sensory bias, sexual selection

## Abstract

Evidence suggests that female preferences may sometimes arise through sensory bias, and that males may subsequently evolve traits that increase their conspicuousness to females. Here, we ask whether indirect selection, arising through genetic associations (linkage disequilibrium) during the sexual selection that sensory bias imposes, can itself influence the evolution of preference strength. Specifically, we use population genetic models to consider whether or not modifiers of preference strength can spread under different ecological conditions when female mate choice is driven by sensory bias. We focus on male traits that make a male more conspicuous in certain habitats—and thus both more visible to predators and more attractive to females—and examine modifiers of the strength of preference for conspicuous males. We first solve for the rate of spread of a modifier that strengthens preference within an environmentally uniform population; we illustrate that this spread will be extremely slow. Second, we used a series of simulations to consider the role of habitat structure and movement on the evolution of a modifier of preference strength, using male color polymorphisms as a case study. We find that in most cases, indirect selection does not allow the evolution of stronger or weaker preferences for sensory bias. Only in a “two-island” model, where there is restricted migration between different patches that favor different male phenotypes, did we find that preference strength could evolve. The role of indirect selection in the evolution of sensory bias is of particular interest because of ongoing speculation regarding the role of sensory bias in the evolution of reproductive isolation.

## Introduction

“Sensory bias,” under its broadest definition, refers to females responding to particular signals from males because their sensory systems are preadapted to such stimulation (Dawkins and Guilford 1996). This definition includes a broad set of female preferences for male traits that resemble important environmental cues (food, Basolo 1990; Proctor 1991; Clark and Uetz 1992; eggs, Egger et al. 2011) as well as for those male signals that simply exploit response to color, auditory transmission, or movement (Ryan and Rand 1993; Boughman 2002; Seehausen et al. 2008; Tobias et al. 2010). Arnqvist (2006) delineates two possible origins for female sensory bias: adaptive sensory biases, where females have evolved under natural selection to respond to particular stimuli (food, predator avoidance, etc.) and these preferences are a target for novel male traits (i.e., males that mimic food), or hidden preferences, which are not the result of selection but instead rely on the neutral consequences of an organism's physiology. Less well considered is the issue of whether the strength of sensory biases would be expected to evolve in the context of sexual selection. An understanding of current preferences, however, is incomplete unless both origination and subsequent evolution of preference is analyzed. Although female preferences may originate, via sensory bias, by external sources of selection, can sexual selection in these cases promote the strengthening (or weakening) of these preferences?

Fuller et al. (2005) discuss inconsistencies in the empirical literature regarding whether sexual selection modifies female preferences that are under sensory bias. They maintain that sensory bias, by definition, does not evolve by sexual selection but instead via natural selection on ecologically relevant perceptual traits. They present a quantitative genetic framework for the evolution of preferences and traits in which sexual selection on preferences themselves (in terms of a selection gradient on preferences due to variation in mate number or quality, “direct” selection) is absent. We are also not interested in sexual selection acting directly on preference strength. When sexual selection occurs on male traits, however, preference evolution should follow via indirect selection, due to the genetic associations (linkage dis-equilibrium) that form automatically between female preferences and male traits (e.g., Kirkpatrick 1982; note that this requires genetic variation in both the preference and trait). We are interested in the effects that this indirect sexual selection may have on the evolution of the strength of preferences formed by sensory bias, in a variety of ecological contexts.

The strength of sensory biases is of particular interest because of the role that sensory bias has been proposed to play in speciation (Boughman 2002). Environmental adaptation produces and maintains variation in male signaling traits, female sensory systems, or both. When divergence in male traits occurs alongside divergence in preferences, speciation may occur. Boughman (2002) suggested that reproductive isolation could arise as a byproduct of the adaptive divergence of communication systems to different environments (rendering the preference a “magic trait,” wherein a single trait is both under divergent selection and a source of reproductive isolation; see Servedio et al. 2011). These ideas have been further developed for the maintenance of color polymorphisms by divergent natural selection favoring locally adapted visual systems (Gray and McKinnon 2007) and for the adaptive divergence of chemosensory systems (Smadja and Butlin 2009). Evolution of the strength of sensory biases both within a single population and in populations with substructure may influence the probability that speciation based on sensory bias can occur.

We focus our analysis on the case, documented in several empirical studies, in which females have a preference for the more conspicuous of alternate male phenotypes in a given habitat (e.g., Boughman 2001; Leal and Fleishman 2004; Uy and Endler 2004; Gray et al. 2008). We are interested specifically in the evolution of the strength of preference for the conspicuous male, not in changes in what the females perceive as conspicuous. Mechanistically, evolution in our model may therefore best be thought of as acting on a component of preference comprising the behavioral response of females, for example whether or not to accept a male given a certain level of conspicuousness, rather than on the sensory system of the female per se.

We consider the evolution of preferences for conspicuousness in several ecological scenarios. First, we consider a single habitat in which sensory bias has led to a preference for a conspicuous male trait, and ask whether mutations for stronger preferences can spread by indirect selection alone. It is well understood that indirect selection on preferences may often be weak during sexual selection, partly because it relies on the strength of genetic associations between preference and trait loci (see Kirkpatrick and Barton 1997). We use this model to illustrate that within a single population, the selection for modifiers of preference strength is expected to be unusually weak, even given these prior expectations. We next use a series of microhabitat models, based on the work of Chunco et al. 2007, who established conditions for the maintenance of polymorphism in such models, to consider the evolution of stronger (and weaker) preferences for conspicuous males. Preferences for conspicuousness per se is of particular interest in these microhabitat models since males with different traits may be conspicuous in different microenvironments. In each of these models, we begin with a population where male trait polymorphisms and female preferences of a fixed strength are already present. By starting at an initial equilibrium with a male trait polymorphism, maintained via female preferences (as in Chunco et al. 2007), we can ignore transient dynamics involved in determining whether or not polymorphisms are maintained, and instead focus on what happens next in terms of preference evolution. We assume in these models that movement of males and females between microhabitats occurs readily at the natural selection and/or sexual selection stages of the life cycle. Finally, we consider a two-island model in which two populations are locally adapted and gene flow occurs between them. Although it has previously been argued that sensory biases may coevolve with male traits when specific biases are beneficial to females via direct benefits or decreased search costs (Arnqvist 2006), we are interested in the previously unaddressed question of whether or not the indirect selection arising from within the system of preferences and traits alone can act as a force for change in the strength of female preferences. In order to isolate the effects of this indirect selection on preferences, we assume that both direct natural and direct sexual selection on female preferences are absent.

## Model Description

We model the evolution of preference strength during sensory bias using a haploid, two-locus, two-allele population genetic model. Males are polymorphic for a trait with two distinct morphs; although this could be any trait (e.g., visual, auditory, or chemosensory), for convenience we refer to the trait as color. Male color polymorphisms are common in fish (e.g., Seehausen and Schluter 2004; Gray and McKinnon 2007) and lizards (e.g., Sinervo and Lively 1996), and research on the genetic basis of these color polymorphisms has shown that color expression is controlled in large part by a single locus with multiple alleles (in fish, Baer et al. 1995 and Fuller and Travis 2004; in lizards, Sinervo and Zamudio 2001). Thus, we model color as a single locus with two alternate alleles; we refer to these as yellow (*T_Y_* occurring with frequency *t_Y_*), and blue (*T_B_* occurring with frequency *t_B_*). We assume that color is entirely genetically determined and females do not express color. Although male color does not indicate genetic or phenotypic quality, males are subject to natural and sexual selection based on their coloration. The conspicuousness of a male depends on an interaction of his color with the background of the microhabitat that he is currently in. During natural selection, more conspicuous males are selected against (as they are more visible to predators); during sexual selection, female prefers conspicuous males because they are more visible. We assume that males of different colors do not have correlated differences in other traits. To maintain variation in male traits, we assume bidirectional mutation between the two alleles (note that in some of the cases with substructure, variation can be maintained even without this assumption).

Females choose mates according to genetically pre-determined preferences coded at the preference locus. Because we are interested in the evolution of preference strength, the population is initially fixed for an allele determining baseline preference strength, referred to as *M*_1_, which causes a female to be α times more likely to mate with a conspicuous male than a nonconspicuous male if she encounters one of each. We introduce an allele that modifies female preference, *M*_2_, which makes females *αɛ* times more (or less, if ɛ < 1) likely to mate with a conspicuous male if encounter rates are equal. We are interested in understanding when, and if, the preference strength modifier *M*_2_ can spread within the population. The assumptions of our model lead to four genotypes: T_B_M_1_, T_B_M_2_, T_Y_M_1_, and T_Y_M_2_, whose frequencies are referred to as *x*_1_, *x*_2_, *x*_3_, and *x*_4_.

Because this is a model of sensory bias in which females prefer conspicuous males, we assume that preferred (conspicuous) males experience higher predation rates. If, for example, the blue morph is more conspicuous in a certain habitat, then males with the blue allele are selected against during natural selection (because they are more conspicuous to predators), but they are selected for during sexual selection (because they are more conspicuous to females).

Through the following models, we ask whether or not modifiers of the strength of preferences for conspicuous trait may evolve within a population. Further, we seek to determine how habitat structure affects the evolution of these preference modifiers.

## General Model

We first present a model of the evolution of the preference modifier in a single population, to serve as a basis of comparison for our other models. Natural selection occurs, selecting against males with a conspicuous phenotype (blue, for convenience) with strength *s*:


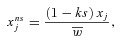
(1)

where 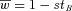 is a normalization that ensures that the genotype frequencies after natural selection sum to 1, and *k* = 1 if *j* = 1 or 2, and *k* = 0 otherwise. The superscript “ns” refers to after natural selection.

Following natural selection, mate choice occurs. Females with an *M*_1_ allele are α times more likely to mate with conspicuous (blue) males, and females with an *M*_2_ allele are *αɛ* times more likely to mate with conspicuous males (or less likely, if ɛ < 1). The proportion of each type of cross is shown in matrix **F** depicted in Table 1. Here, *F_ij_* represents the proportion of matings taking place between an *x_i_* female and an *x_j_* male after natural selection. Female mating frequencies are normalized by *z*_1_ or *z*_2_, defined in Table 1, to ensure that all female genotypes have equal mating success. (Thus, we assume strict polygyny.)

**Table 1:** General model, mate choice

		Males			
		
		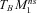	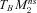	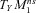	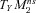
	*T_B_M*_1_	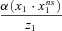	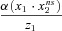	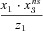	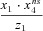
	*T_B_M*_2_	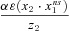	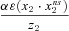	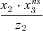	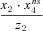
Females	*T_Y_M*_1_	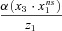	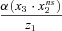	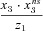	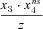
	*T_Y_M*_2_	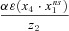	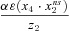	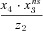	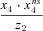
		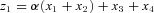			
		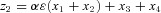			

We assume that free recombination occurs leading to the production of zygotes. As zygotes are formed, bidirectional mutation takes place at a rate 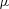 between blue and yellow morphs such that


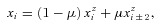
(2)

where in (2), *i* + 2 is used for *i* = 1 or 2 and *i*– 2 is used for *i* = 3 or 4, and 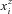 is the genotype frequency in zygotes.

To determine if, and how much, a modifier allele will spread, we can calculate the change in the frequency of *M*_2_ between generations:


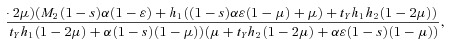
(3)

where


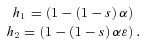


Here, *D* represents the linkage disequilibrium between the color and modifier alleles (*D* = *x*_1_*x*_4_−*x*_2_*x*_3_). In analyzing (3), we are most interested in the case where the frequency of the *T_Y_* allele is nearly 0 or 1; in a single population with an initially fixed preference allele of a given strength maintaining a color polymorphism without mutation is unlikely—generally either natural selection is stronger, favoring the less conspicuous morph, or sexual selection is stronger and favors the more conspicuous morph (see Kirkpatrick 1982). Because of recurrent mutation in our model, we expect low frequencies of the rare morph to remain in the population. The numerator of (3) is scaled by *D* (see also Kirkpatrick 1982), which is by definition very small in our cases of interest where there is very little variation in the trait locus. (The modifier locus also has very little variation when a new modifier allele is introduced at a low frequency, leading *D* to be even smaller.) Because *D* is very small, we conclude that the modifier is unlikely to spread at any significant rate. This result was confirmed via numerical simulations (for example, in the ranges tested, frequencies of the modifier rose by no more than 0.0025 after 10,000 generations).

## Microhabitat Models

To extend our model to additional ecologically relevant sensory bias scenarios, we consider microhabitat population structure, as in Chunco et al. (2007). Individuals live in heterogenous environments and experience microhabitats that differ in physical properties that influence color perception, such as light intensity, substrate color and pattern, or visible spectra. Because of their physical differences, male color is perceived differently in each habitat (e.g., Boughman 2001). In one habitat, henceforth referred to as *H_Y_*, yellow males are more visible—both to predators and potential mates. In *H_B_*, blue males are more visible. The frequency of the habitats is denoted by *h_B_* and *h_Y_*.

Microhabitat ecology, along with sensory bias, has been implicated as an important factor in maintaining male trait polymorphisms (color, Gray and McKinnon 2007; Seehausen et al. 2008; auditory signals, Ryan and Rand 1993; Tobias et al. 2010). Chunco et al. (2007) determined the conditions for polymorphism maintenance under the versions of the microhabitat model we will consider below. We are interested in whether modifiers of preference strength will spread in the each of these scenarios.

As before, the life cycle consists of natural selection, mate choice, and reproduction. However, now that we have microhabitat structure, each step of the life cycle can happen either within a microhabitat or across microhabitats. Because of this, we develop four models: fine-scaled environment (both natural and sexual selection take place across habitats), Levene soft selection (natural selection occurs within habitats, sexual selection occurs across habitats; Levene 1953), local mating (natural selection occurs across habitats, sexual selection occurs within habitats), and a two-island model (both natural and sexual selection occur within habitats, with migration possible in juveniles).

As in the single population model, the more conspicuous morph is selected against during natural selection. We assume that blue males are selected against in habitat *H_B_* and yellow males are selected against in habitat *H_Y_*. Natural selection acting within habitats is equivalent to males remaining in a single microhabitat for the period in which natural selection occurs, for example if males are preyed upon as they remain in a specific microhabitat as juveniles. This also assumes that predators remain in one specific microhabitat at least for each specific prey selection event. In contrast, when natural selection occurs across habitats, we can envision males moving randomly throughout microhabitats between individual predation events, suffering predation in relation to the frequency of habitats.

When natural selection occurs within habitats, the frequency of genotypes in males can be calculated by modifying equation (1); the frequency of genotypes within each microhabitat is calculated, and the population-wide frequency is simply the weighted average of the two habitats: (This assumes that the population density of the focal species between each habitat remains equivalent because of equal densities of predators.)


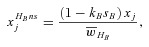
(4a)

where 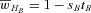 and *s_B_* is the selection coefficient against blue males in habitat *H_B_*, and *k_B_* = 1 if *j* = 1 or 2 and *k_B_* = 0 otherwise, and


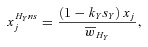
(4b)

where 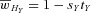, *s_Y_* is the selection coefficient against blue males in habitat *H_Y_*, and *k_Y_* = 1 if *j* = 3 or 4 and *k_Y_* = 0 otherwise. The population-wide frequencies of the genotypes are


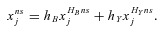
(4c)

When natural selection occurs across habitats, we do not need to calculate genotype frequencies for habitats independently. Instead,


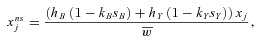
(5)

where 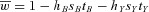 and *k_B_* = 1 and *k_Y_* = 0 if *j* = 1 or 2, and *k_B_* = 0 and *_Y_k_Y_* = 1 otherwise.

Following natural selection, mate choice occurs. Females prefer the more conspicuous male morph; *M*_1_ females are α times more likely to mate with a conspicuous male, while *M*_2_ females are *αɛ* times more likely. In our simulations, we consider both symmetric (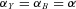) and asymmetric (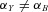) female preferences. Like natural selection, sexual selection can either occur within or between habitats. Sexual selection occurring within habitats assumes that females chose from among males that they see within the microhabitat in which they are present in when they decide to mate. Sexual selection occurring between habitats assumes that females travel between microhabitats at the time of mating, examining males in both before they choose a mate. Within habitats, we consider two independent mating tables, for mate choice within *H_Y_* and *H_B_*: within *H_B_*, mate choice occurs as in Table 1. Within *H_Y_*, mate choice favors the yellow males (see Table 3). Thus, 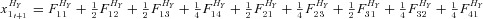
(assuming free recombination, *r* = 1/2). As with natural selection, total offspring frequencies are calculated as weighted averages of the two habitats:


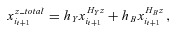
(6)

where, for example, 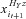 is the frequency of zygotes at time *t*+ 1 in habitat *H_Y_*.

**Table 2:** Listing of variables and definitions used throughout this paper

Variable	Definition
*T_Y_*, *T_B_*	Yellow and blue trait alleles, respectively
*t_Y_*, *t_B_*	Frequency of yellow and blue trait alleles
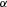	Strength of female preference
*M*_1_	Allele for baseline female preference
*M*_2_	Modifier allele for female preference
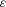	Strength of modification of *M*_2_
*x*_1_, *x*_2_, *x*_3_, *x*_4_	Frequencies of the four male genotypes (T_b_M_1_, T_b_M_2_, T_y_M_1_, T_y_M_2_)
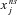	Frequency of male genotype *j* following natural selection
*s*	Strength of natural selection
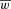	Mean fitness of the population
*F*	Mating table
*F*_*i*,*j*_	Frequency of mating between *x_i_* and *x_j_* parental genotypes
*z_i_*	Normalization term in a mating table to ensure all female genotypes have equal reproductive success
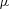	Mutation rate between blue and yellow morphs
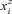	Frequency of the *x_i_* genotype in zygotes
*D*	Linkage disequilibrium
*H_B_*, *H_Y_*	Blue and yellow habitats, respectively
*h_B_*, *h_Y_*	Frequency of blue and yellow habitats
*s_B_*, *s_Y_*	Selection coefficient against blue males in blue habitat and yellow males in yellow habitat, respectively
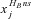	Frequency of *x_j_* genotype in the blue habitat following natural selection
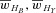	Normalization terms for blue and yellow habitats during natural selection
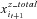	Frequency of *x_i_* zygotes across habitats in the two-patch model
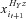	Frequency of *x_i_* zygotes within the yellow habitat in the two-patch model
*m*	Migration rate between blue and yellow habitats
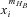	Frequency of *x_i_* genotype in blue habitat after migration
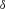	Probability, in the directed movement model, that a male will move to his preferred habitats
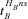	Frequency, in the directed movement model, of blue males in the blue habitat following natural selection
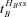	Frequency, in the directed movement model, of blue males in the blue habitat following sexual selection

**Table 3:** Mate choice in *H_Y_*

		Males			
		
		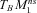	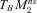	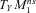	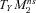
	*T_B_M*_1_	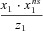	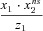	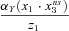	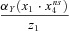
	*T_B_M*_2_	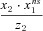	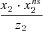	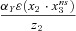	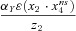
Females	*T_Y_M*_1_	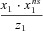	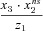	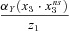	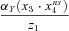
	*T_Y_M*_2_	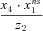	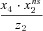	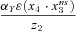	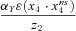
		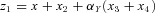			
		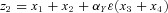			

If sexual selection instead occurs across habitats, female preferences depend on habitat frequency: the probability of being in a habitat where a given male phenotype is more conspicuous determines her preference (see Table 4). As before, following mate choice free recombination occurs to produce zygotes. In all models, bidirectional mutation occurs after zygote production between the *T_B_* and *T_Y_* alleles (mimicking mutation in the gametes), as described in equation (2).

**Table 4:** Across habitat mating table

		Males			
		
		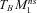	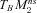	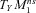	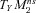
	*T_B_M*_1_	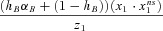	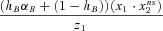	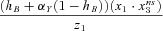	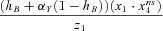
	*T_B_M*_2_	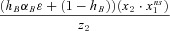	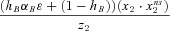	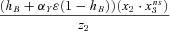	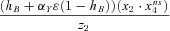
Females	*T_Y_M*_1_	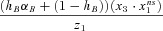	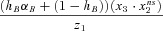	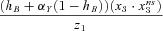	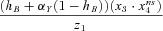
	*T_Y_M*_2_	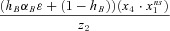	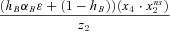	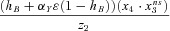	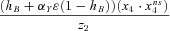
		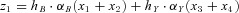			
		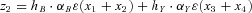			

Each combination of natural and sexual selection ecology was analyzed using numerical simulations in Matlab to determine whether or not male polymorphisms could be maintained (reproducing the results of Chunco et al. 2007), and if so, whether preference modifiers could spread. After evolving to an initial equilibrium in trait frequencies (see Chunco et al. 2007), we introduced the modifier allele *M*_2_ at low frequency (0.1% in the results presented in this paper; other values were tested but we found that this did not impact the outcome of our simulations) and allowed evolution for 15,000 generations to measure its spread. (This number of generations was always found sufficient to determine the alternate outcomes of fixation versus no spread.) If a modifier allele fixed, we introduced successively stronger modifiers to determine if there was a limit to evolvable preference strength. Below, the results from each model are described in detail, and are summarized in Table 5.

**Table 5:** Ecological model results

Model	Natural selection	Sexual selection	Male polymorphism	Preference modifier spread
Fine-scaled environment	Across	Across	No	N/A
Local mating	Across	Within	**Yes**	None*
Levene	Within	Across	No	N/A
Two island	Within	Within	**Yes**	**Rapid**
Directed movement	Selection for camouflage	Selection for mates	**Yes**	None*

* Some variation in the modifier was maintained through mutation selection balance, but it evolved too slowly to be substantial.

### Fine-scaled environment model

In the fine scaled environment model, we consider a situation where both natural and sexual selection occur across microhabitats. For this model, we used equation (4) and Table 3 to model the life cycle. This scenario is analogous to animals that move frequently between microhabitats throughout their life cycle (perhaps due to small patch size), and thus experience the selective pressures of both habitats in relation to their frequency. As in the general model, we found that this did not maintain polymorphisms beyond the small bit of variation produced through mutation (except in the special case where *h_Y_* = *h_B_* and *s_Y_* = *s_B_*; see Chunco et al. 2007) and as a consequence, preference evolution is not a consideration in this case.

### Local mating model

In the local mating model, individuals experience natural selection throughout their lives as they move between habitats, as in equation (4) but then find mates within microhabitats (following Tables 1 and 3). Behaviorally, this is equivalent to gravid females deciding that it is time to mate, and choosing from among males who are locally available wherever the female happens to be. (Males are still assumed to be distributed randomly between microhabitats when mating begins.) This scenario, the primary one examined by Chunco et al. (2007), preserves male polymorphisms. The introduction of a preference modifier served only to change the equilibrium frequency of male morphs very slightly (due to the perturbation of the introduction), but did not spread further.

### Levene soft selection

The Levene model had the opposite assumptions of the local mating model—individuals remain in their microhabitats during natural selection, but mates are chosen across habitats. This is also equivalent to a species where lek mating occurs, and individuals meet in a central location to choose mates. This model leads to the loss of male polymorphism (again with the exception of a small amount due to mutation; see Chunco et al. 2007).

### Two-island model

Our two-island model is formally equivalent to the scenario typical of secondary contact between two populations that are locally adapted to different habitats. Individuals are subject to both natural and sexual selection within habitats (see eq. 1 and Table 1) with bidirectional mutation as in equation (2). Migration between habitats occurs in juveniles (i.e., after mutation and zygote formation):


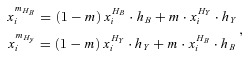
(6)

where *m* is the migration rate between habitats, and 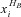
is the frequency of zygotes of genotype *i* in habitat *H_B_* following mating. 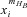 then replaces *x_j_* in equation 4(a), and natural selection acts on males within patches. Mating occurs within patches, following Tables 1 and 3, recombination occurs, and the life cycle repeats.

**Figure 1:**
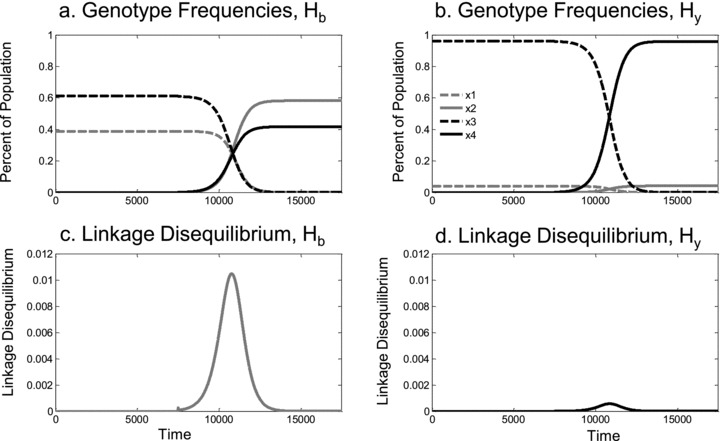
Within-patch allele frequencies and linkage disequilibrium in the two-island model. This figure shows the simulation results from the two-island model with starting conditions *a_Y_* = *a_B_* = 3, *s_B_* = *s_Y_* = 0.25, *m* = 0.1, and *h_B_* = 0.75. Panels a and b show allele frequencies within the two habitats (blue and yellow) before and after the modifier is introduced. With this degree of habitat asymmetry, in the blue patch, the blue phenotype (gray lines) makes up the majority, while in the yellow patch, nearly all individuals are yellow (black lines). In both patches, the modifier rapidly fixes after it is introduced (dashed line—original preference, solid line—modified preference). Panels c and d show linkage disequilibrium in both patches—as the modifiers are fixing, linkage forms between the blue allele and the modifier in the blue patch, and between the yellow allele and the modifier in the yellow patch.

**Figure 2:**
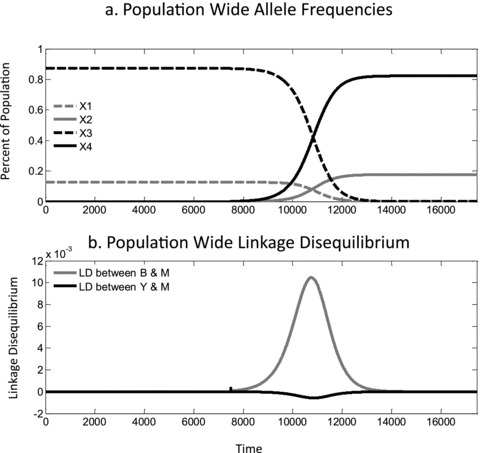
Global allele frequencies and linkage disequilibrium in the two-island model with initial conditions *a_Y_* = *a_B_* = 3, *s_B_* = *s_Y_* = 0.25, *m* = 0.1, and *h_B_* = 0.75. The top panel, a, shows global allele frequencies before and after the modifier is introduced, where the ancestral genotypes are designated by dashed lines and the genotypes with the modifier are designated by solid lines. When preference strengths are changed, the equilibrium frequency of the blue (gray lines) and yellow (black lines) morphs change, but polymorphism is maintained and the modifier fixes. The bottom panel, b, shows global linkage disequilibrium between the blue allele and the modifier (gray) and yellow and the modifier (black). The slight spike around generation 7500 indicates when the modifier was introduced.

We found that as long as migration is present but not so high as to produce a completely mixed population (tested numerically between 0.1% and 49%), male polymorphisms could be maintained. When *m* = 0, the two-island model becomes identical to the general model within each population (and polymorphisms are lost); when *m* = 50 %, the model is identical to the fine-scaled environment model, where both natural and sexual selection occur across patches. When male color polymorphisms were maintained, successive modifier alleles were able to spread; in each habitat, there was continual selection for the more favored allele type and, due to linkage dis-equilibrium between the favored allele and the modifier locus, preference modifiers were able to evolve within habitats (example in Fig. 1). Globally, we found that both male morphs could be maintained and preference modifiers fixed (example in Fig. 2).

We also examined the effects of altering the size of the increase between the initial preference strength and the modifier allele (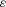), the strength of selection within patches (*s_B_* and *s_Y_*), and the strength of the initial preference (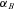 and 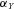). Larger values of 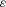 and 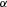 increased the rate of modifier spread, but none of these impacted whether or not the preference allele would spread—as long as selection against, and preference for, conspicuous morphs was present, modifiers could fix.

**Figure 3:**
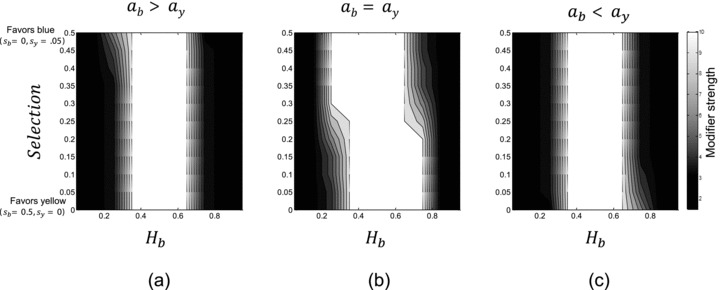
The effect of asymmetry on modifier spread in the two-island model. The *x*-axis is the frequency of blue habitat, going from 0% (all yellow) to 100% (all blue). The *y*-axis shows selection asymmetry, as the values increase, the asymmetry between selection in the two patches increases as follows: *s_Y_* = *y*, *s_B_* = 0.5 −*y*. For example, at 0, *s_B_* = 0.5, *s_Y_* = 0, and at 0.25, *s_Y_* = *s_B_* = 0.25. For this figure, *m* = 0.1 and 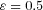. Each panel shows a different preference scenario; in (a), preference is strength for the blue morph with *a_B_* = 10, *a_Y_* = 5; in (b), preference is equal, with *a_B_* = *a_Y_* = 5. And in (c), yellow is preferred more strongly than blue with *a_B_* = 5, *a_Y_* = 10. The shading indicates the final value of *M*_2_ that was able to fix in the population going from black (no modifier fixed) to white (10 successive modifiers fixed; simulation terminated at this point).

When conditions are asymmetric (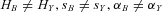), the spread of the modifier alleles can be damped (Fig. 3). As long as polymorphisms are present, at least one modifier allele can fix despite asymmetric selection strengths and habitat frequencies. However, the range of successive modifiers that are able to spread is moderated by asymmetry—in scenarios where one morph is much favored over the other, or one habitat is much more common than the other, there is a limit to the eventual preference strength that can evolve. Figure 3 shows the combined effects of unequal selection, habitat asymmetry, and asymmetric initial preference strengths: each panel shows habitat frequency, from all *H_Y_* through all H_B_, on the *x*-axis, and selection strength, ranging from favoring the yellow morph to favoring the blue morph, on the *y*-axis, and each panel displays the results of preference evolution under different preference regimes. In 3a, the initial preference for blue is greater, in 3b, preferences are equal, and in 3c, preference for yellow is stronger. Comparing across the three panels, we see that increased asymmetry, from any source, can either prevent polymorphism from evolving (black regions) by favoring one morph too strongly over the other, or damp the spread of modifier alleles (gray regions). When all three parameters (habitat, natural selection, and sexual selections) favor one morph, predictably, polymorphism is lost (3a and 3c).

Finally, we examined the potential for preference loss via the spread of a modifier for weaker preferences, that is 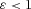. In these cases, we found that in the two-island model such modifiers failed to spread, although recurrent mutation produced some insubstantial variation. When a modifier increases preference strength for the more visible male phenotype in a given microhabitat, linkage disequilibrium between the modifier and the male color allele forms and allows the spread of the modifier. In the case of a modifier for weaker preference, this linkage disequilibrium would lead to the loss of the modifier instead.

### Directed movement

Finally, we considered the possibility of directed movement, where males could choose to move to the habitat where they suffered reduced predation during natural selection, and then move to the habitat where they were more attractive to females when they were ready to mate. Thus, after natural selection, the frequency of blue males in *H_B_*will be 
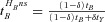
, and the frequency of blue males in *H_Y_* will be 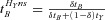, where 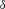 represents the probability that a male will select the correspondingly colored habitat. Prior to sexual selection, males move to the habitat where they are more visible, such that after natural selection the frequency of blue males in *H_B_* will be 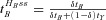, and the frequency of blue males in *H_Y_* will be 
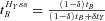
.

These assumptions allowed the maintenance of polymorphisms under a broad range of environmental conditions, as long as males were likely to select the correct habitat (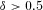) but preference modifiers again failed to spread beyond mutation/selection balance.

## Discussion

We set out in this study to determine the evolutionary pressures placed on the strength of female preferences during sensory bias not from natural selection on the sensory system but from the action of sexual selection that arises from sensory bias itself. Selection placed on female preferences from within a Fisherian system such as this one is indirect; natural and sexual selection changing male trait frequencies leads to changes in the frequencies of female preference (and/or preference modifier) alleles due to linkage disequilbrium between trait and preference loci. We considered several ecological scenarios, all characterized by the assumption, consistent with sensory bias, that different male phenotypes were conspicuous in specific habitats in which females (and predators) preferred them. We found that under the majority of scenarios examined, indirect selection will not lead to any notable amount of evolution of preference strength when preferences arise from sensory bias. This is true for the evolution of preference strengths within a single population, in a variety of microhabitat models, and when males can direct their movement to minimize predation events and maximize their chances of being chosen as a mate. The only scenario in which preference strength can evolve is when the biological scenario falls under the assumptions of a two-island model. The reason for these differences in the ability for modifiers of preference strength to spread, as we describe below, has to do with the ability for linkage disequilibrium to build up between traits and modifiers of preference strength in the different scenarios examined.

In a single population, a modifier of preference strength evolving by indirect selection alone is expected to evolve slowly, due to the fact that indirect selection on preferences, and by extension modifiers of preference, in sexual selection models is generally weak (Kirkpatrick and Barton 1997). We illustrate that in the situation of a modifier of a preference for conspicuousness in a sensory bias scenario, this effect is exaggerated by the fact that linkage disequilibrium is likely to be unusually low. Specifically, when there is sensory bias within a single population, preference (as opposed to the lack of any preference) is likely to be initially fixed; if preferences are a product, for example, of the physiology of the sensory system, they will likely be present in all females. The initial conditions of this scenario will generally result in the subsequent fixation of one of the trait alleles in this two-locus system. Although we altered this condition in our model by allowing bidirectional mutation at the trait locus, so little variation in the trait is maintained that it is impossible for significant linkage disequilibrium between a trait and modifier of preference strength to build, especially when the modifier is introduced at a low frequency. A modifier of preference strength will therefore not spread in a realistic time frame under these conditions. In natural populations, however, significant trait variation is often present despite the existence of female preferences. We caution therefore that under natural conditions the spread of a modifier may be somewhat greater than found here, although it will still be limited by the fact that the strength of linkage disequilibrium will be low if the modifier is initially rare.

In the microhabitat models considered, there is an even more fundamental reason why modifiers of preference strength will not spread, namely that linkage disequilibrium will not build between the trait and modifier loci. When there is microhabitat structure, female preferences under sensory bias are not simply present or absent based on a genetic cue (e.g., consistently for a specific male trait), rather they are always for the more conspicuous male phenotype, which is determined by an interaction of the trait genotype with the environment. In other words, the male trait in this system can be thought of not as just color, but as the property of conspicuousness itself. Thus, nonrandom mating causes a genetic association to be formed between the modifier of preference strength and a different trait allele within different microhabitats. Because at some point in the life cycle in these models the male phenotypes reassort randomly between microhabitats, this temporary linkage disequilibrium dissolves. The scenario of sensory bias thus inherently prevents the buildup of linkage disequilibrium in these types of microhabitat models, and modifiers of preference strength cannot spread.

Only in the two-island model does linkage disequilibrium between the color and modifier loci evolve, and only in that case because individuals do not move between habitats enough to break up the linkage disequilibrium that builds—females in the yellow habitat, for example, always prefer yellow males over blue, and because their offspring remain in that habitat, sufficient linkage disequilibrium builds over generations for the modifier to spread. (The same is true in the blue habitat.) If individuals in our simulations were to be allowed to migrate freely between habitats, linkage dis-equilibrium would be lost and preference modifiers would fail to spread further. Interestingly, polymorphism maintenance and the evolution of preference strength are more robust to high migration rates in this model than in two-island models of reinforcement (e.g., Servedio 2000). In contrast to a traditional model of reinforcement, our models have no frequency-dependent sexual selection (i.e., the frequency of traits in females does not affect the direction of their preferences); natural and sexual selection are always acting in opposite directions in the patches, as determined by the property of conspicuousness, thus preventing loss of variability in the male trait.

Although we only found increases in preference possible in the two-island model, it is important not to discount this possibility. We found modifiers spreading when migration was below 50%—although this is technically a “two-island model,” that is something of a misnomer. Typically, migration between “island” populations is very low, for example, 1% (Pinho and Hey 2010). Our models support the spread of modifiers with much higher contact rates, which could potentially correspond to scenarios of parapatry and mosaic sympatry (Mallet et al. 2009).

It is important to remember, however, that the goal of these models was to identify evolutionary forces on preferences during sensory bias that arise through indirect selection specifically; these are not likely to solely determine the fate of modifier alleles in nature. It is possible, for example, that there may be direct selection against modifiers that increase preference due to the fact that stronger preferences sometimes cause females to incur greater search costs (e.g., Alatalo et al. 1988; Hedrick and Dill 1993; Gotthard et al. 1999; see also Real 1990; Reynolds and Gross 1990). It is also expected that if preferences are set by sensory biases, the natural selection that initially established the preference is likely to still be acting. This leaves open the possibility that such selection may also affect preference strength. Either of these sources of selection would act directly on female preference; such direct selection may be substantially stronger than indirect selection (Kirkpatrick and Barton 1997). We found very little to no indirect selection on modifiers of preference strength in our single population and microhabitat models; in these cases, there is no force to counteract any direct selection that may be present on preferences in the system. We would expect that in the two-island model, the indirect selection that we found on modifiers of preference strength will combine with any existing direct selection to determine the ultimate strength of preferences.

Our models imply that speciation involving sensory bias is unlikely to be facilitated by the indirect evolutionary forces that sensory bias itself places on preference strength, provided that sensory bias simply generates general preferences for conspicuous phenotypes. Sensory bias is most likely to be involved in speciation when preferences diverge due to divergent natural selection on the underlying sensory system (e.g., Boughman 2002). If such preferences were to be strengthened, speciation may be more likely. We note that this is not true when the preference is simply for conspicuousness itself. We found in our microhabitat model that modifiers of preference strength will not spread in this scenario. Even if preferences were infinitely strong, however, speciation would not result in our microhabitat scenarios because, as described above, there is no possibility for linkage disequilibrium to build between preferences and traits; since different males are preferred in different environments and males and females both assort randomly between microhabitats during the life cycle, no isolation is possible. In our two-island model, we find that stronger preferences can evolve. Once again, however, females with a strong preference for conspicuous males will shift the specific male trait allele that they prefer if they are in a different environment, precluding speciation by the Biological Species Concept (Mayr 1942). Finally, even in allopatric populations, our single population model shows that selection generated by sensory bias itself has a very limited effect of the spread on modifiers of preference. True isolation involving sensory bias cannot therefore rely on simply strengthening a preference for conspicuous males, but must instead involve genetic changes that will cause preferences not to change when females relocate (e.g., Van Doorn et al. 1998).

Finally, we would like to emphasize that in addition to finding that the indirect forces generated by sensory bias are unlikely to cause preferences to strengthen, we also found that they cannot decrease the strength of sensory biased preferences. Once present, these preferences will not be lost even if other selective forces on them are removed, in the scenarios modeled here.

To sum, when we isolate the evolutionary forces on the evolution of preference strength that arise during sensory bias, we find that this indirect selection is only likely to be notable when migration is restricted at all points in the life cycle, as in a two-island model. In such scenarios, it would be interesting to examine in more detail whether indirect selection will alter the strength of preferences from an optimum set by natural selection on preferences. Within a single population or in microhabitat scenarios, however, our results suggest that preference strength will not be altered by the action of sensory bias itself.
